# Hydrogels for Antitumor and Antibacterial Therapy

**DOI:** 10.3390/gels8050315

**Published:** 2022-05-19

**Authors:** Xiuling Fang, Cheng Wang, Shuwen Zhou, Pengfei Cui, Huaanzi Hu, Xinye Ni, Pengju Jiang, Jianhao Wang

**Affiliations:** 1 School of Pharmacy, Changzhou University, Changzhou 213164, China; fangxl1998@163.com (X.F.); wangc90@cczu.edu.cn (C.W.); zhoushuwen@cczu.edu.cn (S.Z.); cuizy1990@cczu.edu.cn (P.C.); huhuaanzi@cczu.edu.cn (H.H.); 2 Second People’s Hospital of Changzhou, Nanjing Medical University, Changzhou 213003, China

**Keywords:** hydrogel, antitumor, antibacterial

## Abstract

As a highly absorbent and hydrophobic material with a three-dimensional network structure, hydrogels are widely used in biomedical fields for their excellent biocompatibility, low immunogenicity, adjustable physicochemical properties, ability to encapsulate a variety of drugs, controllability, and degradability. Hydrogels can be used not only for wound dressings and tissue repair, but also as drug carriers for the treatment of tumors. As multifunctional hydrogels are the focus for many researchers, this review focuses on hydrogels for antitumor therapy, hydrogels for antibacterial therapy, and hydrogels for co-use in tumor therapy and bacterial infection. We highlighted the advantages and representative applications of hydrogels in these fields and also outlined the shortages and future orientations of this useful tool, which might give inspirations for future studies.

## 1. Introduction

Hydrogels as systems with a three-dimensional spatial network structure, which use water as their dispersion medium, are soft, can maintain a certain shape, and have a high water absorption capacity (water content can be up to 99%) [1,2]. In addition, hydrogels have good biocompatibility, biodegradability, etc. They are widely used in biomedical fields, such as drug release [3], medical dressings [4], bone repair [5], etc. Hydrogels can be broadly classified into conventional hydrogels and environmentally responsive hydrogels, which have properties such as injectability, self-assembly ability, self-healing ability, etc., and some of them can even respond to specific signals [6,7,8]. Hydrogels are often used in clinical practice, which are made from hyaluronic acid, fibrin, collagen, gelatin, alginate, hydroxyethyl cellulose, carboxymethyl cellulose, poly(hydroxyethyl methacrylate), poly(2-hydroxypropyl methacrylate), poly(acrylic acid) or poly(ethylene glycol), etc. [9]. Hydrogel materials are promising for biomedical applications because of their advantages. (1) They can mimic the three-dimensional environment of the extracellular matrix. Therefore, drugs stored in hydrogels can take advantage of the three-dimensional environment for sustained release and full functionalities. (2) With good flexibility and biocompatibility, the wet environment slip-and-stretch state of hydrogels can effectively avoid secondary injury to patients due to wound adhesion, so they can be used as medical dressings. (3) Cells or biomolecules can be delivered to patients in a minimally invasive manner to improve the treatment efficacy.

It is well known that cancer is a global health challenge. Over the next 20 years, the number of new cancer cases worldwide is expected to increase by approximately 50%. In 2020, more than 19 million people worldwide have been diagnosed with cancer and nearly 10 million have died from it. By 2040, the total number of new cases and deaths are expected to reach approximately 28 million and 16 million, respectively. Cancer treatment alone costs the world about USD 1.2 trillion per year, accounting for nearly 2% of the global gross domestic product in 2019 [10]. The current treatment modalities for tumors include surgery, chemotherapy, radiotherapy, and immunotherapy, among which drug therapy remains the most widely used treatment modality for all types of tumors. However, in clinical practice, the efficacy of monotherapy is often limited. Whether it is traditional cytotoxic drugs or molecular targeted therapies, drug resistance, a lack of efficacy, and tumor recurrence remain huge challenges. However, current combination therapy results based on the combination of conventional agents for administration are not satisfactory due to the short half-life of chemotherapeutic agents and poor tumor selectivity [11]. Systemic drug delivery inevitably leads to the distribution of antitumor drugs in normal tissues and is likely to result in insufficient accumulation of drugs at the tumor site in solid tumors [12]. At the same time, systemic drug delivery is associated with serious side effects, such as nausea, anorexia, and hair loss.

As with cancer, bacterial infections are a global health challenge. Bacterial infections are a common clinical condition with a high prevalence in the clinical setting. Wound healing can be broadly divided into four phases, namely hemostasis, inflammation, proliferation, and remodeling [13]. The prolonged wound healing in most cases is due to bacterial infection at the wound site during the inflammatory phase. The most obvious symptoms of bacterial infection in wounds are redness of the skin, local swelling, increased temperature, and wound pain. As the infection worsens, the bacterial infection at the wound site increases and the patient develops symptoms of pus flowing from the wound. In the treatment of bacterially infected wounds, although novel treatments such as phototherapy (photothermal and photodynamic therapy) and non-antibiotic antimicrobial drugs (nanoenzymes, metallic nanoagents, antimicrobial peptides, etc.) are now also available [14,15,16,17], antibiotics are currently the most used in clinical practice. However, when applying antimicrobial drugs or phototherapeutic to wounds, their loss in the wound reduces the antimicrobial effect, and in some cases they can even be toxic to the tissue for their own reasons. Whether it is a bacterial infection in a skin wound or a bacterial infection associated with cancer, antibiotics are used to treat bacterial infections, which has led to an increasing problem of bacterial resistance [18,19]. Therefore, many researchers are working on the development of antibacterial materials that can overcome bacterial resistance.

**Figure 1:**
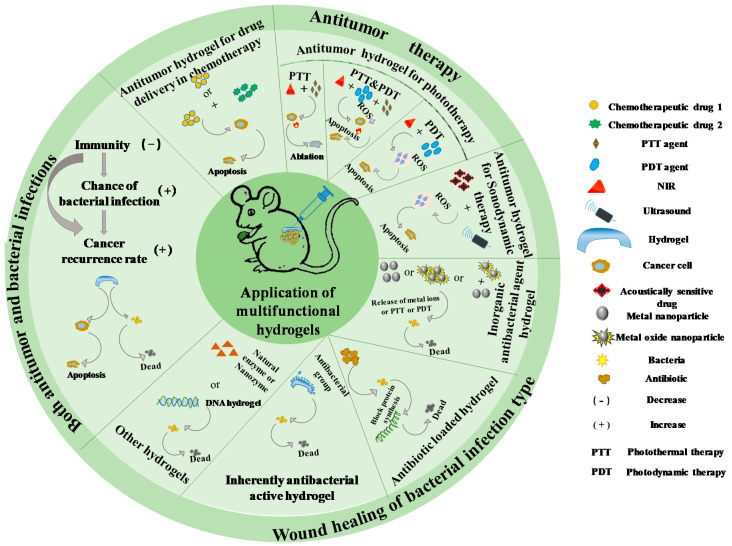
Schematic diagram of the application of multifunctional hydrogels.

In these cases, the local drug delivery of hydrogels is likely to be an effective alternative to systemic delivery of oncology drugs, and can reduce the chance of bacterial resistance. This review classifies the recently developed hydrogels into the hydrogels for antitumor therapy, the hydrogels for antibacterial treatment, and the hydrogels used together for both antitumor and bacterial infections, based on their application in the corresponding therapies (Figure 1). As shown in Table 1, we also briefly classified the functional components of the hydrogels in this review.

## 2. Hydrogel for Anti-Tumor Therapy

### 2.1. Antitumor Therapeutic Hydrogel for Drug Delivery in Chemotherapy

Chemotherapy is currently one of the main methods of tumor treatment, which can be used to inhibit or kill tumors by acting on different stages of tumor cell growth and reproduction. Chemotherapeutic drugs can be classified into alkylating agents, antimetabolites, anticancer antibiotics, botanicals, hormones, and miscellaneous, according to their sources and chemical structures. Chemotherapeutic drugs of the alkylating agent class act directly on DNA, causing DNA damage and preventing the re-growth of cancer cells, such as nimustine and lomustine [20]. Antimetabolites are used in tumor therapy by interfering with the synthesis of nucleic acids, such as 5-fluorouracil [21] and methotrexate [22]. While anticancer antibiotics are widely used in cancer treatment, as a class of microbial cultures extracted from microorganisms, they can interfere with transcription by directly damaging DNA or embedding it. Examples include doxorubicin (DOX) [23] and bleomycin (BLM) [24]. Traditional chemotherapeutic drugs have the disadvantages of rapid metabolism, poor bioavailability, and suboptimal drug absorption and distribution. However, with the study of hydrogels in local tumor therapy, hydrogel-loaded chemotherapeutic drugs allow for a long-term slow release of chemotherapeutic drugs in situ.

**Figure 2:**
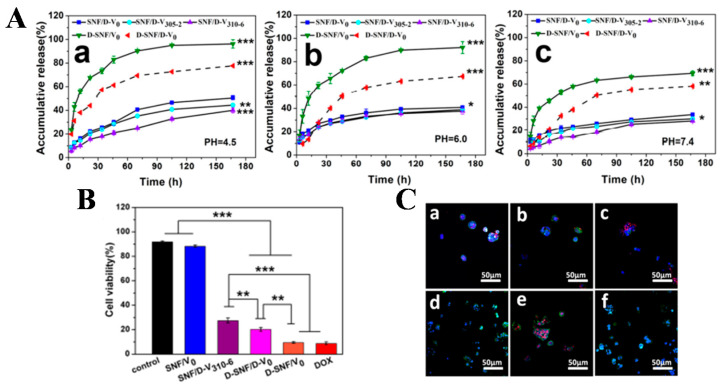
(**A**) pH-dependent release of DOX from the SNF/D-V_0_, SNF/D-V_305-2_, SNF/D-V_310-6_, DSNF/V_0_, and D-SNF/D-V_0_ systems for 3−167 h at pH 4.5 (**a**), 6.0 (**b**), and 7.4 (**c**). Statistically significant: * *p* < 0.05, ** *p* < 0.01, *** *p* < 0.001; (**B**) viability of human breast cancer cells (MCF-7) cultured in vaterite−silk hydrogels and DOX-loaded vaterite−silk hydrogels. Statistically significant: ** *p* < 0.01, *** *p* < 0.001; (**C**) morphology of human breast cancer cells (MCF-7) cultured in vaterite−silk hydrogels and DOX-loaded vaterite−silk hydrogels. (**a**–**f**) SNF/D-V_310-6_, D-SNF/V_0_, D-SNF/D-V_0_, DOX-free silk−vaterite microspheres (SNF/V_0_), free DOX, and control TCP groups (MCF-7). Reprinted with permission from Ref. [27]. Copyright 2019, American Chemical Society.

DOX, which is widely used in clinical practice for cancer treatment, belongs to the anthracycline antibiotics and prevents RNA transcription by embedding and binding to DNA, and also prevents DNA replication. In this way, DOX causes apoptosis of tumor cells. Youngbum Yoo et al. designed a visible light-cured glycol chitosan-based hydrogel loaded with DOX for the treatment of thyroid cancer, which was called GC10/DOX [25]. The rapid and then slow release behavior of DOX in GC10/DOX allowed for a long-term slow release of DOX in tumor tissues, exhibiting better antitumor effects than free DOX. However, during the GC10/DOX formation, the possible limitations caused by some potentially toxic substances, such as cross-linked monomers and photoinitiators in vivo, should also be considered. These toxic substances may affect the activity of loaded bioactive drugs or damage normal organs. Therefore, hydrogels with good biocompatibility, adjustable degradability, and maintenance of drug function are of particular importance. Silk is a natural biomaterial with special properties that can form a variety of morphological structures for local and systemic drug delivery, such as scaffolds, films, hydrogels, fibers, foam balls, capsules, microneedles, etc. Moreover, silk has good biocompatibility, biodegradability, and low immunogenicity [26]. Thus, DOX was effectively loaded in a synergistic silk–vaterite hydrogel delivery system for antitumor therapy. Vaterite microspheres with tunable stability were introduced into the silk nanofiber hydrogel to form a synergistic drug carrier system (Figure 2). This system solved the problems of poor water dispersion of vaterite microspheres and poor drug release from silk nanofiber hydrogels without affecting the injectability of the hydrogels [27]. Other chemotherapeutic agents loaded in hydrogels for antitumor treatment can also show good anticancer effects. For example, methotrexate (MTX)-loaded hydrogel can inhibit the growth and reproduction of tumor cells by preventing DNA synthesis through the inhibition of dihydrofolate reductase by MTX [28]. Researchers designed a CH-β-GP-CNT heterogeneous hydrogel to load MTX [29]. The hydrogel was highly biocompatible. β-glycerophosphate (β-GP) acted as a neutralizing agent to promote the formation of thermosensitive CH-β-GP hydrogels from chitosan, allowing the mixture of chitosan and β-GP to form a hydrogel at body temperature (37 °C) and a solution state at room temperature (25 °C). The researchers doped carbon nanotubes (CNTs) into the hydrogels to strengthen the mechanical strength. Meanwhile, it was found that CNTs could improve the delivery of the therapeutic agent MTX and enhance the anti-tumor effect of MTX.

It has been shown that at least two drugs acting together at the tumor site in chemotherapy treatment facilitate tumor killing, which results in a reduced dose of chemotherapy drugs, fewer side effects, and a better prognosis [11]. Researchers used β-cyclodextrin to generate a β-cyclodextrin curcumin (CD-CUR) inclusion to improve the physicochemical properties of natural curcumin (CUR), which was poorly soluble and unstable under physiological conditions, and then synthesized a PLGA-PEG-PLGA thermosensitive hydrogel as a way to co-deliver DOX and CD-CUR to the tumor site [30]. The PLGA-PEG-PLGA hydrogels loaded with DOX and CD-CUR were injected around the tumor to form gel+DOX+CD-CUR gels in situ. The sustained release of CUR and DOX from the gel at the tumor site caused DNA damage as well as ROS generation. Thus, the dual delivery system of gel+DOX+CD-CUR could improve the cytotoxic efficiency of DOX and promote tumor cell apoptosis. In addition, Tefera Worku Mekonnen et al. used injectable in situ-formable sodium deoxycholate hydrogel (Na-DOC-hyd) to control the release of DOX and RESV (resveratrol) at the pH of the tumor extracellular microenvironment, which then reduced the exposure toxicity of DOX to normal tissues [31].

### 2.2. Antitumor Hydrogel for Immunotherapy and Radiotherapy

Immunotherapy for tumor is a treatment method used to activate immune cells in the body, as well as to enhance the body’s anti-tumor immune response through immunological methods, restart and maintain the tumor-immune cycle, restore the body’s normal anti-tumor immune response, and thus control or clear the tumor [32]. In the immune checkpoint blockade (ICB) therapies, ICB antibodies are designed to block the tumor’s immune evasion mechanisms, and these antibodies can activate the patient’s anti-tumor immune system by inhibiting other signaling pathways [33]. Nitric oxide (NO) is an endogenous gas that has a dual relationship with tumors: low concentrations of NO promote tumor growth, while high concentrations of NO have anti-tumor effects [34]. The systemic administration of ICB therapy may cause severe immune adverse reactions, so Jihoon Kim et al. prepared a thermosensitive hydrogel system using gelatin and Pluronic^®^ F127 for the controlled release of S-nitrosoglutathione (GSNO) as an NO donor and an antibody blocking immune checkpoint cytotoxic T-lymphocyte-associated protein-4 (anti-CTLA-4) to achieve antitumor immunotherapy [35]. This orthogonal immune network regulated by NO enhanced the efficacy of ICB therapy and further strengthened the antitumor effect. To improve the immune response to ICB therapy, an injectable supramolecular hydrogel (HUD hydrogel) was synthesized by polymerizing hyaluronic acid methacrylate (HAMA) with 2-(3-(6-methyl-4-oxo-1,4-dihydropyrimidin-2-yl)ureido)ethyl methacrylate (UPyMA) and diethylene glycol methacrylate (DEGMA) [36]. HUD hydrogels were used for the delivery of DPPA-1 peptide and DOX. In this hydrogel system, DOX was used as a drug to induce immunogenic cell death (ICD) in cancer cells, killing tumor cells while activating the antitumor immune response by inducing immunogenic cell death. DPPA-1 peptide, a D-peptide antagonist with a high binding affinity to programmed cell death ligand 1 (PD-L1), could enhance T-cell-mediated immune responses by blocking the PD-1/PD-L1 pathway. This injectable supramolecular hydrogel exerted a synergistic antitumor effect while reducing the chance of systemic side effects of the drug. Microwave ablation is the direct puncture of microwave needles to the tumor site, where polar molecules in the tissue move at high speed under the action of microwave fields, generating heat by friction with each other and inducing complete denaturation and necrosis of cancer cell proteins thereby inducing cancer cell death [37]. Yuting Cao et al. first combined microwave ablation with immunotherapy for antitumor treatment. The hydrophobic immune adjuvant imiquimod (R837) was added to sodium alginate hydrogel to form an injectable RA hydrogel, which had good microwave sensitivity and biocompatibility and induced efficient antitumor immunity while improving the effect of microwave ablation, effectively inhibiting tumor metastasis [38].

Radiotherapy (RT) for tumors is the application of ionizing radiation or radioactive substances to treat tumors, such as alpha, beta, and gamma rays produced by radioisotopes and X-rays, electron rays, proton beams, and other particle beams produced by various types of X-ray therapy machines or gas pedals to treat malignant tumors [39]. Radiation resistance is one of the reasons for the limited efficacy of RT and is related to the tumor microenvironment, such as rapid value-added and hypoxic environment [40]. O_2_ can assist ionizing radiation to induce DNA damage and thus apoptosis, while hypoxia can lead to resistance due to compromised efficacy of radiotherapy [41]. Endostatin (ES) is an endogenous anti-angiogenic peptide that enhances the anti-tumor effect of RT by inhibiting tumor neovascularization to improve the efficiency of oxygen delivery [42]. However, ES is mainly administered systemically, which can lead to systemic toxic effects. Therefore, Na Wang et al. mixed ES into hyaluronic acid tyramine to form ES/HA-Tyr hydrogel, which could release ES slowly and prolong the half-life of ES while reducing the systemic toxic effects of ES [43]. The combination of ES/HA-Tyr hydrogel with RT effectively inhibited the survival of tumor neovascularization, reduced hypoxia at the tumor site, and improved the antitumor effect of RT. It was also studied that gold nanoparticles were added to sodium alginate hydrogel along with the anticancer drug cisplatin as a radiosensitizer to form an ACA nanocomplex to enhance the antitumor effect of RT [44]. The tumors of the mice treated with ACA+RT for 28 days were all reduced in size and all survived two months later, and the ACA+RT treatment could reduce the clinical dosage of anticancer drugs and X-rays to ensure the antitumor effect while minimizing the toxic side effects of the treatment. Some chemotherapy or radiotherapy methods induce the development of ICD to activate anti-tumor immunity, which will be enhanced by the presence of immune adjuvants [45]. Lele Sun et al. first combined sodium alginate with ATP-specific aptamer (Aapt) and then hybridized it with the immune adjuvant CpG oligonucleotide (CpG ODN), which belongs to single-stranded DNA, to form ALG-Aapt/CpG [46]. Additionally, ALG-Aapt/CpG could form hydrogels in situ by intratumoral injection. When hydrogel was applied to oxaliplatin chemotherapy or X-ray radiotherapy, the dead tumor cells would release adenosine triphosphate (ATP) due to ICD, which then competitively bound with Aapt and caused the release of CpG ODN, thus allowing the anti-tumor immunity to be further enhanced. ALG-Aapt/CpG hydrogel could improve the anti-tumor effect of chemotherapy or RT and inhibit tumor metastasis by activating an anti-tumor immune response as well as an immune memory effect.

### 2.3. Antitumor Hydrogel for Phototherapy

Phototherapy refers to the irradiation of the lesion area by a light source, especially by a near-infrared light source, to stimulate phototherapeutic reagents to kill tumor cells for therapeutic purposes. The two main forms of phototherapy include photodynamic therapy (PDT) [47] and photothermal therapy (PTT) [48]. Among the most extensively studied drug delivery for phototherapy are nanoparticles, which can enhance the solubility of photosensitizers [49] and photothermal agents [50]. However, the targeting ability of phototherapeutic agents is usually poor because they are distributed throughout the body. To solve this problem, various hydrogel drug delivery platforms have been developed to deliver antitumor drugs and phototherapeutic agents for antitumor therapy, which can achieve the effect of “one injection, multiple phototherapies”.

#### 2.3.1. Antitumor Therapy Hydrogel for PTT

**Figure 3:**
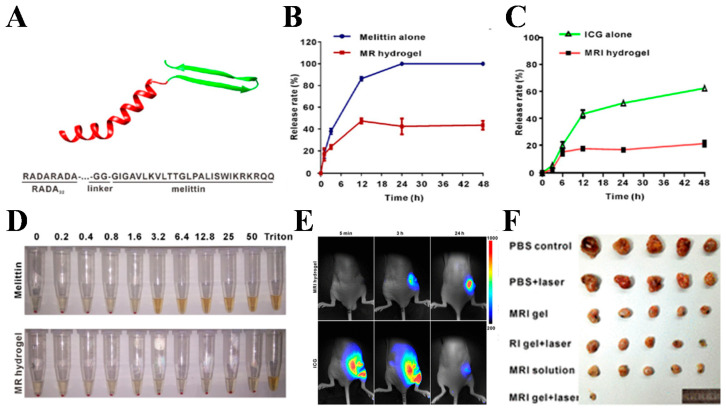
(**A**) Diagram of the molecular structure of the RADA_32_−melittin fusion peptide; (**B**) comparison of the melittin release profile between the MRI hydrogel and melittin solution over a period of 2 days; (**C**) comparison of the ICG release profile between the MRI hydrogel and ICG solution over a period of 2 days; (**D**) evaluation of the hemolysis effect of the MRI hydrogel and free melittin; (**E**) fluorescence imaging of MRI hydrogel and ICG solution biodistribution at 5 min, 3 h, and 24 h post-intratumoral injection; (**F**) evaluation of the in vivo antitumor efficacy of the MRI hydrogel. Reprinted with permission from Ref. [54]. Copyright 2017, American Chemical Society.

PTT is a treatment modality that uses materials with high photothermal conversion efficiency to kill cancer cells by converting light energy into heat energy. The photothermal gel, which is gathered near the tumor tissue, is used to kill cancer cells when the photosensitizer is excited by specific wavelengths of light to generate heat under the irradiation of an external light source. Based on the combined anti-cancer therapy of NIR-induced thermotherapy and vascular disruption, Yuqing Liang et al. synthesized an injectable nanocomposite (NC) hydrogel using Prussian blue (PB) nanoparticles, combretastatin A4 (CA4), and gellan gum, which had tumor-suppressive ability due to significant tumor site retention and the sustained release of CA4 [51]. The hydrogel provided an “attack + protection” anti-tumor strategy, in which PB-based NIR irradiation exerted a strong attack on most cancer cells, while CA4 prevented tumor growth by cutting off the energy supply. The photothermal efficiency of PB in gellan gum was enhanced. The disruption of tumor vasculature and the prolonged release of CA4 ensured the synergistic therapeutic effects. It is a major pursuit that designing biocompatible and effective anticancer biomaterials to achieve relatively low tumor recurrence rates in cancer photothermal therapy (PTT). For example, Melittin (bee venom peptide), the main component and primary bioactive substance of bee venom, is a cationic peptide composed of 26 amino acid residues. Based on its powerful membrane-disrupting ability, Melittin is an effective anti-cancer drug. However, when used alone, Melittin is hemolytic in vivo [52,53]. Therefore, a novel cytotoxic hybrid and ICG-containing MRI peptide hydrogel with melittin in the backbone and ICG in the matrix was developed for enhanced PTT treatment of glioblastoma [54]. The MRI peptide hydrogel reduced the hemolysis rate of Melittin and induced sufficient heat to kill tumor cells in the presence of NIR light irradiation, enhancing the anticancer efficacy and providing excellent in vivo biocompatibility. Thus, it provided an effective alternative tool for PTT application in tumor therapy (Figure 3). In addition, Ankit Gangrade et al. developed a photoactive nanocomposite filament-based drug delivery system, which exhibited the in vivo on-demand release of DOX [55]. The single-walled carbon nanotubes equipped with functional modifications of DOX were encapsulated in free filament hydrogels. The increased thermal and electrical conductivity of nanocomposite hydrogels was due to the electric field responsiveness and the NIR laser-induced superheat effects exhibited by nanocomposite filamentous hydrogels, which allowed for the on-demand release of the contained drugs. The nanocomposite hydrogel was applied to the tumor site in a non-invasive manner. The antitumor effect of the hydrogel without cardiotoxicity was observed after regular stimulation of the nanocomposite hydrogel by the simultaneous or separate application of an electric field and an NIR laser. Xiaolin Hou et al. also combined PTT, chemotherapy, and immunotherapy for the treatment of breast cancer in which the PC_10_ARGD peptide was first synthesized, and then Ag_2_S QDs, DOX, and the immune adjuvant Bestatin were integrated into the PC_10_ARGD peptide to form an injectable Ag_2_S QD/DOX/Bestatin@PC_10_ARGD hydrogel. The hydrogel system exhibited good photothermal properties due to the presence of Ag_2_S QDs, which accelerated the release of DOX from the hydrogel, and Bestatin enabled the hydrogel to elicit a strong immune response by enhancing the function of T lymphocytes. Therefore, Ag_2_S QD/DOX/Bestatin@PC_10_ARGD hydrogel could inhibit tumor growth and tumor metastasis [56].

#### 2.3.2. Antitumor Therapy Hydrogel for PDT

PDT relies on the irradiation of a specific wavelength light source to activate the production of biotoxic reactive oxygen species (ROS) in the tumor tissue, which in turn oxidizes and damages the tumor and activates anti-tumor immunity. Hypoxia, a common feature of the tumor microenvironment, reduces the immunogenicity of tumors by weakening the function of cytotoxic T cells and attracting regulatory T cells. It also exacerbates tumor cell genetic instability and activates some tumor survival factors, thus causing tumor tolerance to chemotherapy and radiotherapy and promoting tumor metastasis [57,58,59,60]. Therefore, the tumor hypoxic microenvironment is closely related to tumor development, prognosis, and metastasis, and the effectiveness of treatment. Tian-Jiao Zhou et al. put forward an extended oxygen-producing phototherapy hydrogel (POP gel) system to be used for the inhibition of tumor spread and the growth of primary tumors [61]. They used Pluronic^®^ F127 and Pluronic^®^ F68 to prepare the hydrogel loaded with photosensitizers. The introduction of CaO_2_ and catalase (CAT) into the hydrogel system could regulate the hydrolytic reactivity of CaO_2_ by limiting the penetration of water molecules into the hydrogel system to achieve long-term oxygen delivery. Thus, the POP gel could be used for suppressing the growth and spread of the tumor by ameliorating the PDT immunogenic treatment mediated through tumor hypoxia. The photosensitizer chlorin e6 (Ce6) is a second-generation photosensitizer that could be synthesized from demagnetized chlorophyllic acid. The biological activity is similar to that of demagnetized chlorophyllic acid with high efficiency in the production of singlet oxygen [62]. Ce6 exhibits hydrophobicity and aggregates easily in solution. Most of the current studies immobilize it with nanoparticles or couple it with nanoparticles [63,64,65,66]. However, there are still problems such as poor tissue permeability. Karuppusamy et al. prepared a Fu/AL@GG hydrogel using rock algae polysaccharide, alginate, and gellan gum, which was used to encapsulate Ce6 to form a Ce6-Fu/AL@GGH hydrogel system [67]. This hydrogel system caused a large accumulation of Ce6 in tumor cells, which made increased the efficiency of Ce6 to inhibit HT-29 cell growth via PDT. Ce6-Fu/AL@GG hydrogel could be used as a viable vehicle for the Ce6-assisted PDT to selectively eliminate colon cancer cells. A degradable hyaluronic acid hydrogel platform with injectable and NIR light-triggered ROS was also investigated [68], where the HA-ADH hydrogel was prepared by modifying hyaluronic acid with dihydrazide adipate. The water-insoluble photosensitizer protoporphyrin was then chemically coupled to the HA-ADH hydrogel to form a water-soluble HA-ADH-PpIX hydrogel, which was then used as a vehicle for the locoregional delivery of anticancer drug adriamycin (as referred as DOX) to enable a combination of chemico-photodynamic therapy and light-adjustable on-demand drug release. Under laser irradiation, the ROS produced from the hydrogel not only enhanced the PDT effect, but also made the hydrogel degrade through the cleavage of the cross-linker, thus allowing the on-demand release of DOX. Thus, the biodegradable hydrogel could act as an appealing local vehicle for prolonged coupled chemico-photodynamic cancer therapy, which had minimal side effects.

#### 2.3.3. Antitumor Hydrogel for PTT/PDT Combination Therapy

PTT can ablate tumors by using the heat generated by the excitation of photothermal nanoparticles. However, high temperature can lead to overexpression of heat shock proteins to reduce apoptosis and increase tumor recurrence, thus weakening the PTT effect. PDT can enhance the photothermal effect by using reactive oxygen species to eliminate the heat shock proteins produced during PTT. Conversely, PTT can use thermal energy to stimulate the molecular movement of tumor cells and increase oxygen levels around tumor cells, thus alleviating the oxygen-dependent problem of PDT and enhancing its effect [69]. Yuxin Wang et al. developed a new drug delivery system for the photochemotherapy of oral squamous cell carcinoma (OSCC), which was called human serum albumin–indocyanine green cisplatin nanoparticles (HSA-ICG-DDP-NPS) [70]. The system improved the therapeutic efficacy against OSCC compared to single anticancer drugs. However, there were still problems such as poor tissue permeability and systemic distribution, as mentioned above. To combine PTT/PDT combination therapy with hydrogels, Chanjuan Liu et al. proposed a near-infrared modulated thermosensitive hydrogel (PNT gel) consisting of a photothermal network with supramolecular cross-linked conjugated polymers to enhance the stability of ICG [71]. The PNT gel exhibited a gel–sol reversible upper critical solution temperature (UCST) which was marginally higher than body temperature. Thus, it could be carried out to turn the controlled release of ICG on or off by thermally induced gel–solution transition using NIR, effectively eliminating 4T1 cells. In addition, combination treatment had been shown to be effective in enhancing photodestruction without tumor recurrence compared to the treatment with PTT or PDT alone. Gold nanorods (AuNRs) are classical photothermal materials used for PTT and have strong absorption in the near-infrared region (NIR). Its absorption can be modulated and used for combined PTT and PDT by controlling the aspect ratio [72,73]. Gold nanorods (AuNRs) and methylene blue (MB) were encapsulated into gellan gum hydrogels (GGH) using a simple cooling method to construct synergistic cancer phototherapy hydrogels. This hydrogel had good cytocompatibility and excellent PTT/PDT combination for antitumor therapy [74]. Gang Wang et al. also developed injectable and degradable calcium alginate (Ca^2+^) hydrogel for the local delivery of Au/Ag nanorods (Au/Ag-NRs) and adriamycin hydrochloride (DOX.HCl) [75]. The restriction of Au/Ag-NRs and DOX.HCl via ALG hydrogel effectively prevented adverse effects on healthy organs, while long-term retention of therapeutic agents in the tumor was achieved. NIR laser irradiation could kill most of the tumor cells, while triggering the orderly release of DOX.HCl from the hydrogel, thus completely eradicating the tumor. To prevent the efficacy of antineoplastic drugs from being further limited by the inherent multidrug resistance (MDR) of cancer tissue, the positive charge nucleic acid hydrogels (DNA gel) with high permeability, as well as photothermal, injectable, and bio-degradable properties, were synthesized for the delivery of anti-cancer drugs [76]. DNA gel enhanced the clearance of tumor masses. DNA gel therapy remarkably decreased drug resistance and increased survival in mice with in situ mammary tumors compared to DOX chemotherapy alone.

### 2.4. Antitumor Hydrogel for Sonodynamic Therapy (SDT)

**Figure 4:**
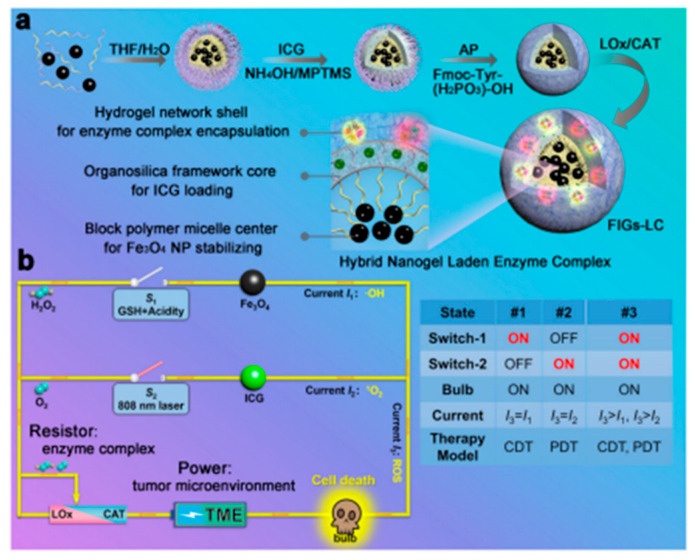
Schematics of synthesis and therapeutic mechanism of FIGs-LC. (**a**) The synthetic procedures of FIGs-LC; (**b**) schematic circuit diagram for the peroxisome-inspired therapeutic mechanism of FIGs-LC based on the dual-enzyme-regulated ROS generation with GSH and NIR activation. Reprinted with permission from Ref. [79]. Copyright 2021, Nature Publishing Group.

Sonodynamic therapy takes advantage of the strong penetrating ability of ultrasound on biological tissues, especially focused ultrasound, which can concentrate acoustic energy to deep tissues without trauma, activating some acoustically sensitive drugs (e.g., hematoporphyrin) to produce free radicals (reactive oxygen species, ROS) with acoustic and cytotoxic properties to kill tumor cells [77]. However, the inadequate provision of oxygen within solid tumors can make SDT much less effective. Therefore, some researchers blended catalase (CAT) with the acoustic sensitizer meso-tetrakis(4-carboxyphenyl)porphyrin (TCPP) into chitosan (CS) and disodium β-glycerophosphate (GP) to develop a thermally triggered in situ hydrogel [78]. In situ sol–gel conversion then occurred by thermal triggering, allowing the hydrogel to be retained at the tumor site. The hydrogel generated ROS under ultrasound treatment and also triggered the breakdown of endogenous H_2_O_2_ to produce O_2_. The generated O_2_ could reverse the hypoxia in solid tumors and further enhanced the efficacy of SDT. Thus, the repeated use of SDT after injection of this hydrogel could provide excellent therapeutic efficacy, leading to effective tumor eradication. Furthermore, Xing Qin et al. combined sonodynamic therapy (SDT), photodynamic therapy (PDT), and chemodynamic therapy (CDT) for antitumor therapy [79]. Lactate oxidase (LOx) and catalase (CAT) were integrated into Fe_3_O_4_ nanoparticles–indocyanine green (ICG) co-loaded with hybrid nanogels (named as FIGs-LC). Through the catalytic metabolism of LOx and CAT in hydrogels, the production of ·OH and ^1^O_2_ could be regulated for glutathione (GSH)-activated CDT and NIR-triggered PDT. The high ROS levels which resulted from the regulated response of FIGs-LC caused lethal damage to cancer cells and triggered effective inhibition of tumor growth (Figure 4).

## 3. Hydrogel for Antibacterial Treatment

### 3.1. Hydrogel for Wound Healing of Bacterial Infection Type

Except for the hydrogel with its own antibacterial activity, most of the hydrogel is still used as a carrier of antibacterial drugs, and this hydrogel itself has no antibacterial effect, or the antibacterial effect is negligible. At this point, the antibacterial drug loaded in the hydrogel is responsible for killing bacteria, while the hydrogel plays a role of a dressing and is effectively fixed on the wound surface, reducing the chance of wound infection while keeping the wound moist and accelerating wound healing.

#### 3.1.1. Inorganic Antibacterial Agent Hydrogel for Wound Healing

Inorganic antibacterial agents take advantage of the antibacterial ability of metals or metal oxides such as silver, copper, and zinc, which are loaded into hydrogel dressings by physical adsorption and agitation. The metallic elements mainly act as antibacterial agents in their ionic form, partly in the form of metal oxides. In environments with relatively high metal ion concentrations, the presence of high concentrations of metal cations outside the microbial membrane alters the normal polarization state inside and outside the biological membrane. This causes a new ion concentration difference, which hinders or disrupts the transportation of small and large molecules required for the maintenance of cellular physiology [80]. However, the antibacterial mechanism of metal antibacterial agent is not very clear, as there are several claims. (1) Metal or metal oxide surfaces dissolve metal ions and combine with the functional proteins in the organism-related reactions to kill bacteria. (2) Metal or metal oxide surfaces generate reactive oxygen species (ROS) caused by oxidative stress, leading to bacterial death. (3) Metal or metal oxide nanoparticles can reduce the ATP level by inhibiting ATPase activity by disrupting the cell wall and cell membrane to induce bacterial death. (4) Metal and metal oxide nanoparticles can mechanically disrupt bacterial cell structure, as irregular protrusions and sharp edges can mechanically disrupt and kill bacteria [81,82,83,84,85,86]. However, the minimum inhibitory concentration of metal antibacterial agents is higher and the solution-based metal antibacterial agents are easily lost so that they do not stay well in the trauma. However, combining the metal antibacterial agent with a hydrogel dressing allows the metal antibacterial agent to effectively stay in the affected area. The use of a lower concentration of metal antibacterial agent and a slower release will still show a strong bactericidal effect.

**Figure 5:**
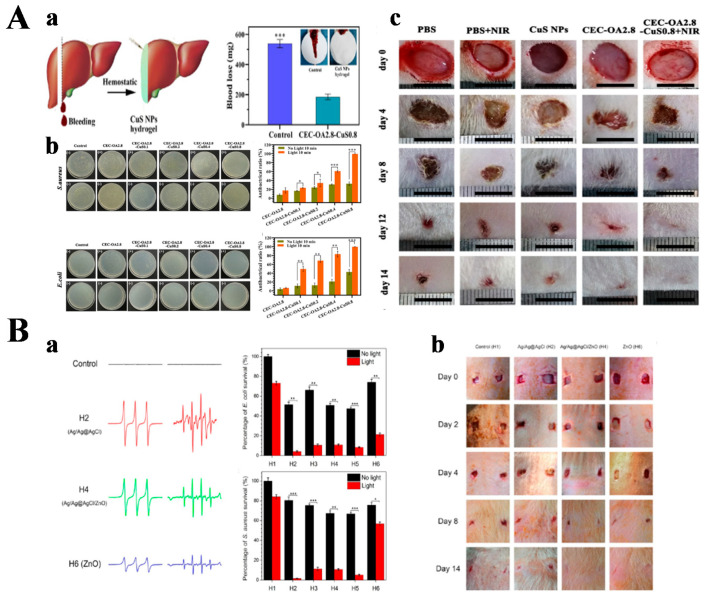
Inorganic antibacterial agent hydrogel for wound healing. (**A**) The CuS NP hydrogel. (**a**) Schematic diagram of preparation of a liver bleeding model and hemostatic property of CuS NP hydrogels; (**b**) antibacterial ability of CuS NP hydrogel; (**c**) in vivo healing evaluation of infected wounds. Statistically significant: * *p* < 0.05, ** *p* < 0.01, and *** *p* < 0.001. Reprinted with permission from Ref. [97]. Copyright 2021, American Chemical Society. (**B**) The Ag/Ag@AgCl/ZnO nanocomposite hydrogel. (**a**) Identification of ROS was detected by ESR spectroscopy and significant enhancement effect on antibacterial activities (H1: control hydrogel; H2: Ag/Ag@AgCl hydrogel; H3, H4, and H5: Ag/Ag@AgCl/ZnO hydrogels, and H4 for representative; H6: ZnO hydrogel). Statistically significant: * *p* < 0.05, ** *p* < 0.01, and *** *p* < 0.001. (**b**) in vivo study on the effects of treatment of *S. aureus*-induced wound infections by hydrogels and the corresponding wound photographs of the rats at days 0, 2, 4, 8, and 14. Reprinted with permission from Ref. [104]. Copyright 2017, American Chemical Society.

Inorganic antibacterial agents have the advantages of high temperature resistance, fast sterilization, and good effect. Among many metal ions, mercury, silver, cadmium, copper, and zinc have antibacterial ability, but the relatively safer use is limited to silver, zinc, and copper ions. Zinc and copper have some antibacterial properties, but their antibacterial strength is only 1/1000 of that of silver ions [87,88]. Therefore, silver resides as the dominant inorganic antibacterial agent, while the application of silver-based antibacterial agents in the biomedical field is mainly in the form of metallic silver, silver salts, and silver nanoparticles. However, the instability of silver nanomaterials can lead to the rapid release of large amounts of Ag^+^, resulting in excessive local cytotoxicity, such that it poses an additional health risk to the human body [89,90,91]. Therefore, it is important to develop new silver-based antibacterial nanomaterials that are efficient, easy to prepare, and capable of safe long-term sustained release of Ag^+^. The high surface energy of ultrasmall Ag NPs (<2 nm), which are highly reactive and strongly cohesive and highly susceptible to oxidation, reduces their antibacterial activity. To overcome these problems, Hanif Haidari et al. proposed to impregnate ultrasmall Ag NPs into F127 thermosensitive hydrogel, which had good biocompatibility [92]. This hydrogel was liquid at low temperature and gel-like when the temperature increased to body temperature. The system allowed for long-term storage and efficient antibacterial activity of the ultrasmall Ag NPs by controlling their release. Compared with the untreated control group, the hydrogel group of ultra-small Ag NPs could effectively remove the biofilm produced by bacteria. It has been shown that to prevent agglomeration of silver nanoparticles, they could be loaded onto two-dimensional materials such as graphene, which could effectively improve the dispersion and stability of silver nanoparticles in solution to enhance their antibacterial effect. As a two-dimensional transition metal sulfide, molybdenum disulfide (MoS_2_) has attracted considerable attention from scientists because of its large specific surface area, excellent photothermal conversion, good antibacterial properties, and biocompatibility [83,93,94,95]. MoS_2_ can be used for PTT and PDT, and polyvinyl alcohol (PVA) is a non-toxic polymer with good water solubility, good biocompatibility, and biodegradability. Therefore, Pengfei Yan et al. prepared a photo-responsive hybrid hydrogel (MoS_2_@PDA@Ag/PVA Hybrid Hydrogel) with good mechanical properties and excellent photothermal antibacterial activity [96]. The researchers combined MoS_2_ with Ag NPs via polydopamine (PDA) to form MoS_2_@PDA@Ag nanoparticles, which were then blended into the PVA hydrogel. The addition of Ag enhanced the photocatalytic performance of the hydrogel. Based on the synergistic effect of PDT and PTT, the hydrogels demonstrated good antibacterial activity under NIR light irradiation. Compared with pure PVA hydrogels, MoS_2_@PDA@Ag/PVA hydrogels could promote wound healing, demonstrating their promising potential for infected wound healing. Copper is one of the essential trace elements in the human body, widely distributed in biological tissues, ranking third after iron and zinc. Cu^2+^ can induce angiogenesis, promote endothelial cell proliferation and migration, and contribute to wound healing. Researchers designed a self-healing injectable hydrogel with hemostatic, antibacterial, and cell-migration-promoting properties [97]. A series of CuS NP hydrogels, which were injectable, self-healing, and had a good photothermal conversion, was prepared based on N-carboxyethyl chitosan (CEC), a derivative of a natural polymer, and sodium alginate oxide (OA) through dynamic imine bonding. These CuS NP hydrogels had good biocompatibility, an excellent hemostatic effect due to the presence of chitosan, and a good antibacterial effect due to PTT action. In addition, Cu^2+^ released from the hydrogels promoted the formation of granulation tissue and the reconstruction of new blood vessels and dermis, thus accelerating the healing of bacterially infected wounds (Figure 5A). Surface plasmon resonance is a unique optical phenomenon exhibited by precious metal nanoparticles (e.g., gold or silver) that leads to the generation of strong electromagnetic fields on the nanoparticle surface, resulting in enhanced radiation absorption and scattering properties. Gold nanorods (Au NRs), as plasmonic nanoparticles, can produce localized surface plasmon resonance (LSPR) effects that can be used for PTT applications [98]. The investigators designed a glycol chitin nanocomposite hydrogel containing a mixture of D-amino acids to facilitate the disruption of complex bacterial biofilms associated with orthopedic implants. Gold nanorods were added to the hydrogel for photothermal treatment to completely eradicate any residual bacteria [99].

Zinc is mainly in the form of ZnO to reflect the antibacterial effect, and there are two main views on the antibacterial mechanism of ZnO nanoparticles [100]. (1) The photocatalytic antibacterial mechanism. Under light irradiation, ZnO can excite oxygen to produce ROS to destroy the proliferation ability of bacterial cells for the purpose of inhibiting or killing bacteria. At the same time, the smaller the particle size of ZnO, the easier it is to generate reactive oxygen species around it. (2) The contact bactericidal mechanism, in which ZnO slowly releases zinc ions in aqueous media. When the zinc ions combine with bacterial cell membranes, the structure of membrane proteins is disrupted to make them inactive for bactericidal purposes. Tingting Wang et al. designed and prepared a zinc oxide/sodium alginate (ZnO/SA) bilayer hydrogel membrane which used ZnO and sodium alginate (SA) as raw materials [101]. The bilayer hydrogel membrane had good antibacterial properties and no significant cytotoxicity. When applied to the skin wounds of SD rats, the ZnO/SA bilayer hydrogel film could more effectively promote wound healing compared with the control group, and the healing effect improved with the increase in ZnO content. The diabetic ulcer is a complication of diabetes, most often found on the patient’s feet, hence the name diabetic foot. In severe cases, it may lead to gangrene and amputation. Xiao Xia Li et al. first prepared Ag-ZnO nanoparticles, and then loaded Ag-ZnO nanoparticles in carboxymethylcellulose/K-hornwort gum/graphene oxide/ konjac glucomannan hydrogel to form Ag-ZnO@CGK hydrogel for the treatment of diabetic foot ulcers [102]. It was shown that the Ag-ZnO@CGK hydrogel had a good antibacterial effect and sufficiently accelerated the wound recovery. Ag-ZnO@CGK hydrogel promoted fibroblast growth and significantly accelerated re-epithelialization. These results demonstrated the incredible potential of Ag-ZnO@CGK hydrogel in wound healing of diabetic foot ulcers. An injectable hydrogel made of folic acid (FA), dopamine (DA), and zinc ions, named DFT-C/ZnO-hydrogel, was prepared by Yiming Xiang et al. [103]. The PDA in the hydrogel was encapsulated around carbon quantum dot-decorated ZnO (C/ZnO) nanoparticles. The rapid production of reactive oxygen species (ROS) and heat under 660 nm and 808 nm light irradiation enabled the hydrogel to exhibit excellent antibacterial effects against both *S. aureus* and *E. coli*. The hydrogel exhibited promising applications in wound repair by continuously releasing zinc ions, thus producing sustained antibacterial effects and promoting the growth of fibroblasts. Other researchers have embedded Ag/Ag@AgCl/ZnO nanostructures into carboxymethylcellulose (CMC) hydrogels. The Ag/Ag@AgCl nanostructures were first assembled in the hydrogel by UV photochemical reduction and then embedded in ZnO nanostructures by NaOH precipitation [104]. This hydrogel system was based on photodynamic therapy for antibacterial activity, with visible light enhancing the production of reactive oxygen species and Ag/Ag@AgCl nanostructures to enhance the photocatalytic and antibacterial activity of ZnO. These enabled the Gram-positive bacteria Staphylococcus aureus and Gram-negative bacteria *Escherichia coli* to be killed rapidly. Furthermore, a large number of leukocytes and neutrophils were produced under the stimulation of continuously released Ag^+^ and Zn^2+^, resulting in a synergistic antibacterial effect and accelerated wound healing (Figure 5B).

#### 3.1.2. Antibiotic-Loaded Hydrogel for Wound Healing

**Figure 6:**
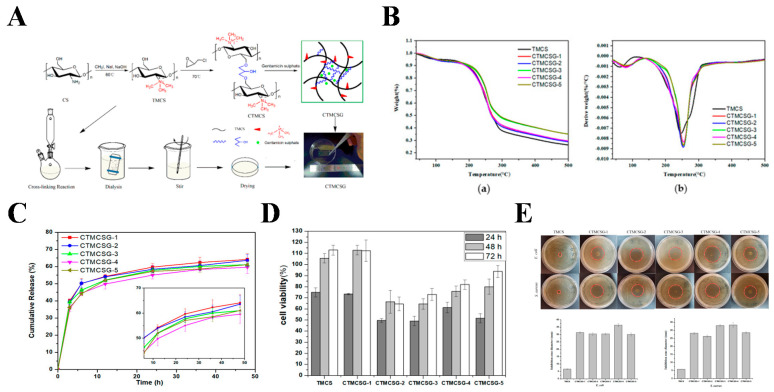
(**A**) Synthesis routes for chitosan hydrogel films; (**B**) thermal Analysis of TMCS film and CTMCSG hydrogel films. (**a**) TGA curves; (**b**) DTG curves; (**C**) the in vitro gentamicin sulfate release of CTMCSG hydrogel films; (**D**) the antibacterial activity of TMCS film and CTMCSG hydrogel films; (**E**) the cytotoxicity of TMCS film and CTMCSG hydrogel films. Reprinted with permission from Ref. [107].

Although there are many researches on various types of antibacterial agents, antibiotics are still the most effective, as well as common antibacterial agents in clinical practice. The clinical antibiotics can be divided into aminoglycosides, amidosteroids, macrolides, and peptide antibiotics. Moreover, the problem of bacterial resistance to antibiotics can be overcome by reducing the dosage of antibiotics, while the antibiotics are loaded in the hydrogel to achieve “small amount and high efficiency” through the slow release of antibiotics locally in the hydrogel. Gentamicin is an aminoglycoside antibiotic, mainly used for the treatment of bacterial infections, especially those caused by Gram-negative bacteria [105]. Gentamicin binds to the 30 s subunit of bacterial ribosomes and blocks bacterial protein synthesis. Researchers have added poloxamer 188 to poloxamer 407 solution to change the gelation temperature of poloxamer hydrogel [106]. The hydrogel was liquid at 25 °C and changed to a gel state at 37 °C. The poloxamer hydrogel was not toxic to L929 cells and the loading of gentamicin inhibited the growth of bacteria from the first hour of testing. Moreover, for similar concentrations of gentamicin sulfate, the poloxamer hydrogel loaded with gentamicin showed a better antibacterial effect than the gentamicin solution. Other researchers have successfully prepared cross-linked hydrogel films with different degrees of cross-linking by the reaction of chitosan quaternary ammonium salt (TMCS) and epichlorohydrin. The CTMCSG hydrogels were formed by loading with gentamicin sulfate [107]. The biocompatibility and antibacterial activity of CTMCSG were determined. The results showed that the CTMCSG hydrogel films were slightly cytotoxic. The zone of inhibition against *E. coli* and *S. aureus* reached about 30 mm, demonstrating its potential value for use as an outstanding antibacterial wound dressing (Figure 6). Chloramphenicol (CAM) is an aminol antibiotic that acts on the 50S subunit of bacterial ribonucleoprotein bodies and blocks protein synthesis. CAM is a bacteriostatic broad-spectrum antibiotic that inhibits both Gram-positive and Gram-negative bacteria [108]. Sveinung G. Ingebrigtsen et al. prepared a hydrogel liposome system for the skin delivery of CAM [109]. Hydrogel liposome systems were formed by double centrifugation (DC) with nanoliposomes mixed into CS hydrogels. The in vitro antibacterial effect of CAM in the hydrogel liposome system was better compared to CAM in solution. Erythromycin belongs to the macrolide class of antibiotics, and its antibacterial mechanism is similar to that of chloramphenicol in that it penetrates the bacterial cell membrane and inhibits bacterial protein synthesis by reversibly binding to the 50S subunit of the ribonucleoprotein body of bacteria in close proximity to the donor site (P-site) [110]. A composite film dressing consisting of pluronic F127-pectin and pluronic F127-gelatin was investigated as a potential wound healing drug delivery system, with 0.1% erythromycin (ER) being integrated into the film. The ER-loaded composite film allowed the controlled release of ER with antibacterial activity against *S. aureus* and no toxic effect on human skin fibroblasts. This suggested that the composite film had potential as an antibacterial wound dressing [111]. Sicheng Yang et al. proposed an injectable micromotor@hydrogel (MM@hydrogel) drug delivery system based on nano/micromotors [112]. The system consisted of a (PSS/PEI)_10_ Janus micromotor and Schiff base hydrogel. Schiff base hydrogel was used as an outer envelope for the micromotor to improve selective release. Antibacterial experiments were performed using MM@hydrogel containing erythromycin, capsule@hydrogel containing erythromycin (bare capsules without platinum nanoparticles loaded into the hydrogel), and Schiff base hydrogel containing erythromycin. The MM@hydrogel containing erythromycin showed the best antibacterial effect. Vancomycin, a glycopeptide antibiotic, interferes with cell wall synthesis by interfering with peptidoglycan which is the key component of the bacterial cell wall structure, thus inhibiting the production of phospholipids and peptides in the cell wall to kill bacteria [113]. One study added vancomycin to oxidize hyaluronic acid (HA) and dihydrazide adipate. The drug release capacity and antibacterial activity were tested [114]. The results showed that after three days, vancomycin had released about 4/5. The hydrogel had good biocompatibility along with excellent anti-biofilm activity. An injectable skin extracellular matrix hydrogel (porcine decellularized skin matrix (ADM)) has also been designed as a scaffold for skin defect repair [115]. This hydrogel had a porous structure that enabled the effective adsorption of vancomycin and rapid release of vancomycin. This showed that the vancomycin-loaded ADM hydrogel was effective in sterilization at the beginning of wound healing. The experiments in rats showed that the hydrogel could effectively control bleeding and avoid vascular damage. Therefore, vancomycin-loaded porcine ADM hydrogel could be used for hemostasis and bacterial infection prevention. Willemijn Boot et al. found that hyaluronic-acid-based hydrogels containing gentamicin and vancomycin performed better than current clinical practice treatments in a large animal model of clinically relevant chronic methicillin-resistant Staphylococcus aureus (MRSA) ODRI, eliminating the bacteria from all animals [116].

#### 3.1.3. Inherently Antibacterial Active Hydrogel for Wound Healing

**Figure 7:**
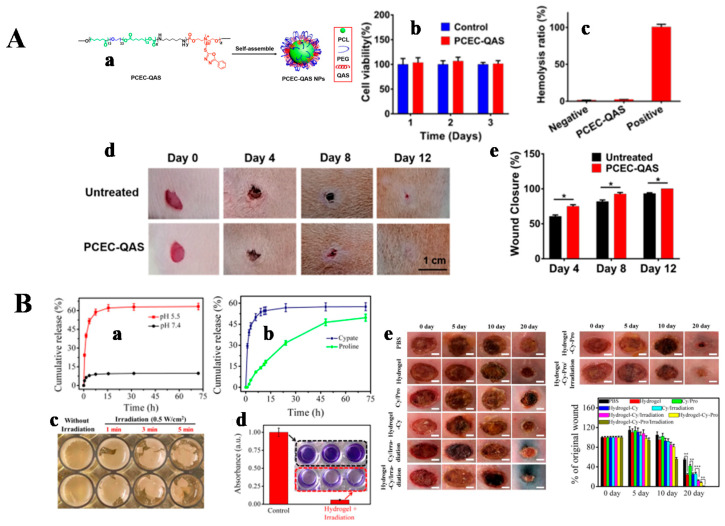
(**A**) The PCEC-QAS hydrogel. (**a**) Schematic diagram of the self-assembly of PCEC-QAS NPs; (**b**) relative viability of 3T3 cells inoculated on the PCEC-QAS hydrogel; (**c**) hemolysis ratio of the PCEC-QAS hydrogel. Negative and positive controls were normal saline solution and distilled water, respectively; (**d**) representative photographs of wounds at day 0, 4, 8, and 12; (**e**) wound closure rates. * *p* < 0.05. Reprinted with permission from Ref. [121]. Copyright 2020, American Chemical Society. (**B**) The IKFQFHFD peptide hydrogel. (**a**) Release curves of cypate loaded in the hydrogel-Cy system under different pH conditions; (**b**) release curves of cypate and proline loaded in the hydrogel-Cy-Pro system (under pH 5.5); (**c**) representative photographs of integrated MRSA biofilm incubated with cypate released from the hydrogel-Cy system at a scheduled time point of 4 h under a NIR laser (808 nm,0.5 W/cm^2^) irradiation for different times; (**d**) crystal violet staining image and its corresponding absorbance for integrated MRSA biofilm incubated with the hydrogel-Cy system (under pH 5.5) for 4 h followed by NIR laser irradiation (808 nm, 0.5 W/cm^2^, 5 min) (the biofilm under NIR laser irradiation without incubation with the hydrogel-Cy system was used as the control); (**e**) in vivo wound healing. Reprinted with permission from Ref. [7]. Copyright 2019, American Chemical Society.

Hydrogels with intrinsic antibacterial ability are becoming one of the main focuses in the field of hydrogel dressings. Many hydrogels with intrinsic antibacterial ability have been developed, including those with antibacterial polymers or antibacterial peptides. A nucleobase-inspired self-adhesive and inherently antibacterial hydrogel wound dressing was synthesized from poly(3-dimethyl(methacryloyloxyethyl)ammonium propane sulfonate)-co-(methacryloylaminoadenine) (PDMAPS-co-PMA-Ade) and chitosan [117]. The hydrogel was reproducibly adherent and antibacterial. 3-dimethyl(methacryloyloxyethyl)ammonium propane sulfonate (DMAPS) conferred stain resistance to the hydrogel, while the interaction between nucleobase-modified methacryloylaminoadenine (MA-Ade) provided molecular recognition of the corresponding groups on the tissue surface. In vivo wound repair results showed no bleeding, reduced inflammation, and decreased neovascularization in the hydrogel-treated mice. The hydrogel promoted wound healing. It was also investigated that the bionic polymer P(GMA-co-MPC) was successfully prepared by atom transfer radical polymerization. Non-toxic CTM prepared P(GMA-co-MPC) hydrogels by chemical cross-linking with epoxy groups in P(GMA-co-MPC) copolymers. The hydrogel exhibited good biocompatibility and inherent antibacterial behavior [118]. The three-dimensional network structure of the hydrogel provided a platform for the loading and release of curcumin (Cur). Wound healing experiments in SD rats showed that P(MPC-co-GMA) hydrogels and P(MPC-co-GMA) hydrogels loaded with curcumin accelerated the wound healing rate. As a new type of intelligent material, self-healing hydrogel has a very wide application prospect in medical and biological fields [119]. Yuan Zeng et al. prepared multifunctional poly(ethylene glycol) (MF-PEG) containing phenylboronic acid and poly(ethylene glycol) terminal phenolic groups by a one-step Ugi reaction. The resulting MF-PEG was able to cross-link poly(ethylene glycol) through dynamic boronic ester bonds and could rapidly generate self-healing hydrogels [120]. This self-healing hydrogel showed antibacterial properties due to the presence of phenolic groups in the MF-PEG unit. Furthermore, the antibacterial ability against *E. coli* and *S. aureus* was similar to that of penicillin–streptomycin combination antibiotics against *E. coli* by 50 times and against *S. aureus* by 10 times of MIC, respectively. Wenshuai Liu et al. developed an inherently antibacterial hydrogel for wound healing of methicillin-resistant Staphylococcus aureus (MRSA)-infected skin by the self-assembly of amphiphilic oxadiazole group-modified quaternary ammonium salt (QAS)-conjugated poly(ε-caprolactone)-poly(ethylene glycol)-poly(ε-caprolactone) (PCEC-QAS) micellar nano-bearing agents [121]. PCEC-QAS hydrogels were fully absorbed with no local or whole body toxicity and could promote wound repair in the absence of drugs or cytokines (Figure 7A). Based on the function of antimicrobial peptide from the current research results, it is generally believed that the antibacterial peptide bactericidal mechanism mainly acts on the cell membrane of bacteria, disrupting its integrity and producing perforation. This causes the cell contents of the bacteria to spill out of the cell, thus killing the bacteria. Jianhao Wang et al. prepared an IKFQFHFD peptide that self-assembles at neutral pH, as well as a supramolecular hydrogel with a pH-adjustable antibacterial nanofiber network for biofilm removal and promotion of wound healing [7]. The antibacterial activity of this peptide hydrogel originated from the instability of nano-fibrous structure at acidic pH and the activated release of IKFQFHFD. Therefore, an additional hydrogel loaded with cyapte and proline was prepared for wound healing. The results showed that the cyapte- and proline-loaded hydrogels exhibited acidic pH-responsive release profiles and could completely eradicate MRSA biofilms with good biocompatibility, facilitating the healing of MRSA-infected wounds in diabetic mice (Figure 7B). Peptide-modified nanofiber-reinforced hydrogels were also designed by Schiffbase dynamic crosslinking [122]. The RRRFRADA peptides were synthesized by the solid-phase synthesis method. The peptide-modified nanofiber-reinforced hydrogel (NFRH) obtained by incorporating nanofibers into the hydrogel greatly enhanced the stability and mechanical strength of the hydrogel, such that the hydrogel could withstand various external forces while restoring its original shape when filling irregularly shaped wounds. NFRH was not only potent for injection and self-healing, but also had possessed inherent antibacterial and hemostatic properties. By inducing blood cell and platelet aggregation, it promoted chronic wound healing. These peptide hydrogel systems provided a therapeutic tool for the treatment of clinical chronic wounds. Xuan Wang et al. also prepared a phototherapeutic antibacterial platform based on peptides (GIIKKIIKKIIKKIGRADARADARADARADA-NH_2_) and copper sulfide nanodots (CuS NDs) for the treatment of bacterially infected wounds [123]. CuS ND-loaded antibacterial peptide hydrogels of CuS ND generated heat as well as reactive oxygen species (ROS) under the irradiation of near-infrared light (NIR), which killed bacteria on the wound surface and promoted wound healing.

#### 3.1.4. Other Hydrogels for Wound Healing

In recent years, enzymes have started to receive attention from researchers as antibacterial agents. In particular, glucose oxidase (GOx), an endogenous oxidoreductase, is widely used in the food and medical fields to prevent bacterial infections. As a biocatalyst, GOx can effectively catalyze the oxidation of β-D glucose to hydrogen peroxide (H_2_O_2_) and gluconic acid, and the resulting H_2_O_2_ has antibacterial properties [124,125]. The presence of a high glycemic environment and bacterial infection delays the healing of diabetic wounds. GOx can demonstrate antibacterial ability in low diabetic wounds while lowering their blood glucose levels. Yuan Zhao et al. loaded GOx in an Ac-Ile-Lys-Tyr-Leu-Ser-Val-Asn-NH_2_ (IKYLSVN) antibacterial peptide hydrogel as a diabetic wound dressing [126]. Tingting Huang et al. embedded GOx and Fe_3_O_4_/TiO_2_/Ag_3_PO_4_ in polyacrylic acid calcium phosphate (PAA-CaPs@Nps@GOx) hydrogel by in situ biomimetic mineralization method [127]. The hydrogel exhibited excellent stability of enzymatic activity and had the advantage of reducing blood glucose concentration and generating ROS, which could effectively promote diabetic wound healing. Since natural enzymes are difficult and expensive to obtain in large quantities in living organisms, there is a growing interest in the research and development of stable and low-cost synthetic artificial mimetic enzymes. Nanozymes, a class of nanomaterials with enzyme-like catalytic activity, are able to catalyze natural enzyme substrates under physiological conditions and follow similar reaction kinetic behavior. This class of enzymes derives its catalytic activity from the special nanostructure of the nanoenzyme itself, without the need to introduce additional catalytic functional groups or natural enzymes [128]. Yang Li et al. prepared a multifunctional PDMoT hydrogel for infected wound healing by incorporating MoS_2_@TA/Fe into PVA/Dex hydrogel [129]. Because of PTT activity, peroxidase-like (POD) activity and the loss of glutathione (GSH), the hydrogel exhibited significant antibacterial activity against *E. coli* and *S. aureus*. The hydrogel could promote cell proliferation and kill bacteria, reduce inflammation, supply oxygen, regulate free radical levels, and facilitate blood vessel growth during the wound healing, which could promote wound healing as well as skin regeneration. Chen Jun et al. also designed a versatile system of a diphenylalanine (FmocFF) hydrogel modified with 9-fluorenylmethoxycarbonyl. It could enhance the oxidase and peroxidase activities of Pt NPs and nanoenzyme materials at a different pH, thus demonstrating excellent antibacterial effects. In this system, hydrogen protons (H^+^) generated by the dissociation of F provide an acidic microenvironment for Pt NPs. As a result, Pt NPs encapsulated in Fmoc-FF hydrogels had 6-fold oxidase-like activity and 26-fold peroxidase-like activity [130]. This versatile system facilitated the development of nanoenzyme hydrogels.

**Figure 8:**
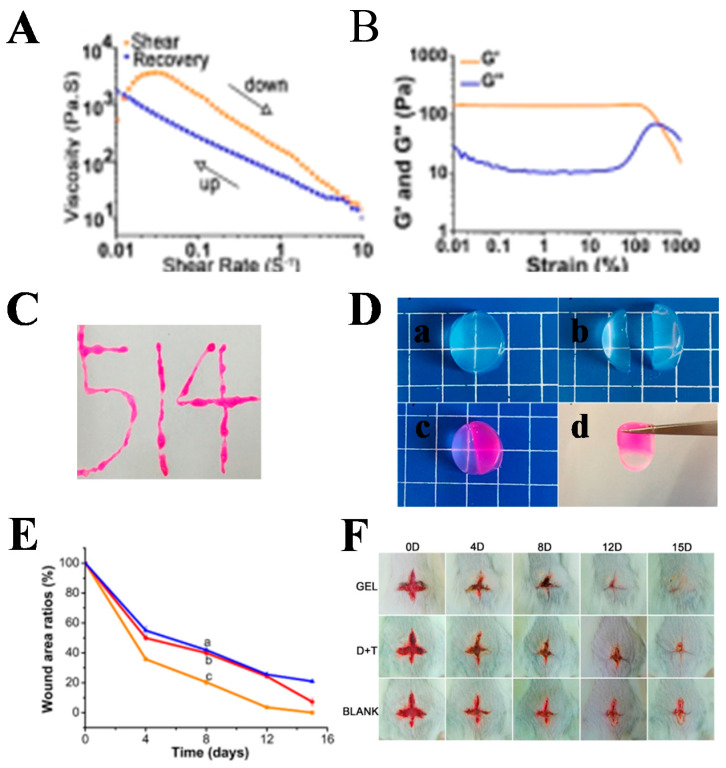
(**A**) Viscosity as a function of shear rate. (**B**) Dynamic modulus of DNT hydrogel under increasing strains from 0.01% to 1000% with a fixed frequency of 1 Hz at 25 °C. (**C**) Injectable property of the hydrogel. (**D**) (**a**) A hydrogel sample was (**b**) cut in half, (**c**) stained with rhodamine (and two fragments were brought together after several minutes), and (**d**) healed into one. (**E**) The wound area was measured by Image Pro Plus and the plot of wound area ratio, from curve a to c, BLANK, (D + T) mixture, DNT hydrogel. (**F**) Photographs of the wounds treated with hydrogels, (D + T) mixture and nontreated at 0, 4, 8, 12, and 15 days, respectively. Reprinted with permission from Ref. [134]. Copyright 2019, American Chemical Society.

DNA hydrogels are hydrogel systems, whereby nucleic acid aptamer acts as the specific detection element, based on the principle of DNA base complementary pairing and nucleic acid aptamer–target molecule interactions. The sol–gel transition properties of DNA hydrogels can be achieved by the reversibility of conformational changes in DNA. DNA hydrogel has both the biological function of DNA and the skeleton function of hydrogel, with good biocompatibility, biodegradability, and structural designability. It has promising applications in the biomedical field [131]. A multifunctional fluorescent DNA hydrogel (RCA-gel) with silver nanoclusters (Ag NCs) as a functional component was successfully synthesized using the relayed RCA method [132]. This hydrogel was synthesized in such a way that it was injectable. It also possesses fluorescent properties and excellent antibacterial activity due to the presence of Ag NCs. RCA gel also showed excellent biocompatibility. The versatility of RCA gel granted other potential applications. A multifunctional DNA hydrogel dressing was prepared by Liping Zhou et al. Based on the principle of base pairing, a dendritic macromolecular X+Y DNA junction structure was synthesized and then dynamically cross-linked with the BPQDs-doped cationic polymer polyethyleneimine (PEI) (PEI@BPQDs abbreviated PB) to form a DNA hydrogel (PBCD gel) with self-healing ability, tissue adhesion, and antibacterial activity [133]. The hydrogels (OPC B2/PBCD gels) had free radical scavenging and antioxidant properties by loading proanthocyanidin B2 (OPC B2). This DNA hydrogel dressing had the ability to convene bone marrow cells to trigger adaptive immunity to facilitate the regeneration of tissue, thereby facilitating wound healing. Xingxing Jiang et al. also synthesized DNT (DNA + THPS) hydrogels with self-healing, shear thinning, and injectability through hydrogen bonding and intermolecular electrostatic interactions between DNA and THPS (Figure 8). DNT hydrogels had broad-spectrum antibacterial ability and good biocompatibility to promote wound healing [134].

### 3.2. Antibacterial Hydrogel for Other Applications

Antibacterial hydrogels are also used for anti-fouling, anti-biofilm, and tissue engineering, in addition to wound healing applications. Microbial contamination of medical materials is a risk for both health care workers and patients [135]. Huan Ding et al. used carboxybetaine ester monomer (CBMAE); acrylamide (AM); N, N0-methylenebisacrylamide (MBA); and ammonium persulfate (APS) as materials to synthesize PVA/PAM/PCBMAE ampholytic double-network hydrogels by physical cross-linking and free radical polymerization methods [136]. The PVA/PAM/PCBMAE double-network hydrogel had favorable electrical conductivity due to the presence of amphoteric ions. The hydrogel could inhibit the growth of *E. coli* while having antifouling properties against bovine serum albumin. It showed a promising application for medical implant materials. Once the bacterial biofilm is formed, it has a natural resistance to antibiotics and the body’s immunity. When the resistance of the body decreases, the surviving bacteria in the biofilm can be released, possibly causing re-infection [137]. A study was conducted to investigate the antibacterial activity and anti-biofilm ability of Ag NPs incorporated into sodium alginate hydrogels [138]. The results showed that the hydrogel could effectively disrupt bacterial biofilms without direct contact with the biofilm to achieve the disruption. Changsheng Du et al. prepared a bionic hydrogel with improved antibacterial efficiency [139]. They used polyisocyanopetide (PIC) and thiadiazolopyridine-thienothiophene-thiadiazolo-pyridine (PTTP) to synthesize the PTTP/PIC hydrogel. The combination of PIC and PTTP enabled PTTP to generate more reactive oxygen species, which improved the therapeutic effect of PDT and thus the antibacterial efficiency. In addition, other researchers used photodynamic antibacterial chemotherapy (PACT) for antimicrobial purposes. Abdechakour Elkihel et al. loaded 5,10,15,20-tetra(1-methylpyridin-4-yl)tetraiodoporphyrin (TMPyP) into xylan hydrogel, which exhibited excellent antibacterial activity when bound to PDT through the long-term sustained release of the photosensitizer TMPyP [140]. Based on previous studies, Yuanxiang Xie’s team loaded lignin–copper sulfide into polyvinyl alcohol (PVA) hydrogels to form LS-CuS@PVA hydrogels [141]. Due to the strong hydrogen bonding between lignosulfonate (LS) and PVA, LS-CuS could be dispersed uniformly in the hydrogel network. Due to the presence of CuS, this hydrogel exhibited excellent antibacterial activity as well as anti-biofilm activity with H_2_O_2_ under the irradiation of near-infrared light with synergistic PTT/PDT/peroxidase-like activity. Hydrogels have long been of interest to researchers for the repair of damaged tissues by providing an extracellular matrix-like environment for the interchange of nutrients or metabolites due to their porous structure [142,143,144,145]. Soyon Kim’s research team used lysozyme and chitosan to synthesize the hydrogel with dual functions of degradability and to control bacterial infection [146]. Furthermore, the hydrogel could coordinate cell action and function by controlling the degradation of the hydrogel matrix, which has promising applications in tissue engineering. Tuyajargal Iimaa et al. considered that hydrogels could be used to make artificial bile ducts; hence, they chose three antibacterial agents (ABAs), including β-Lactam-carbapenem, β-Lactam-cephalosporins, and β-Lactam-carbapenem, which are used in the clinical treatment of biliary tract infections [147]. ABA was cross-linked with gelatin to form a 3D scaffold, and the hydrogel was implanted subcutaneously into rats for evaluation of antibacterial activity and cytocompatibility. The results showed that the hydrogel had excellent antibacterial properties as well as cell adhesion and cell proliferation, and could be applied to tissue engineering. In order to regenerate functional pulp after root canal removal, Maxime Ducret et al. integrated fibrin and chitosan to form the fibrin-chitosan hydrogel [148]. The hydrogel exerted antibacterial activity in vitro due to the presence of chitosan, while fibrin served as a scaffold to promote the proliferation of dental pulp mesenchymal stem/stromal cells (DP-MSC) and their proliferation. However, its application to pulp regeneration needs to be further demonstrated by in vivo experiments. However, Maria Stella Moreira’s team combined chitosan hydrogel with photobiomodulation therapy (PBMT) for pulp regeneration [149]. The experimental results showed that human dental pulp stem cells were able to proliferate and migrate in the chitosan hydrogel combined with PBMT therapy. In vivo experiments in rats showed new pulp-like tissue formation in rats treated with PBMT and chitosan hydrogel via hemagglutination. This suggested a promising application of this hydrogel treatment for pulp regeneration.

## 4. Co-Purpose Hydrogel for Anti-Tumor and Bacterial Infections

**Figure 9:**
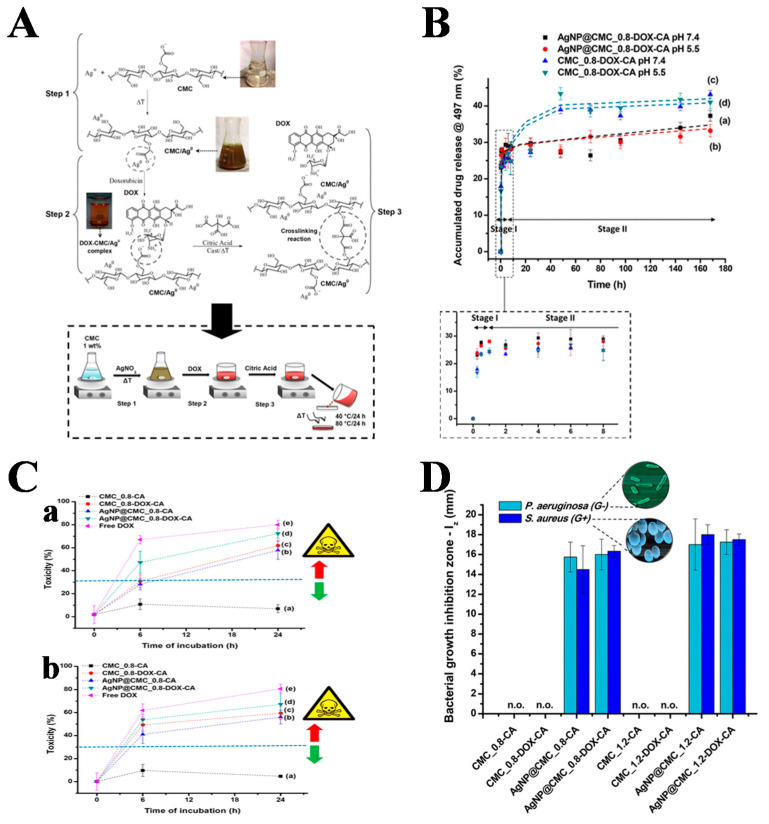
(**A**) Schematic illustration (chemistry perspective) of the complete process of synthesis of the hybrid hydrogel membranes. (**B**) In vitro release of DOX from hydrogels as a function of pH. (**C**) In vitro cytotoxicity based on the MTT assay of (**a**) HEK 293T and (**b**) A375 cell lines after incubation with hydrogels and hybrids. (**D**) Evaluation of resistance/sensitivity of Gram-positive (*S. aureus*) and Gram-negative (*P. aeruginosa*) bacteria toward cross-linked hydrogels: effect of AgNPs, DOX, and AgNPs + DOX (n.o. = not observed). Reprinted with permission from Ref. [155]. Copyright 2019, American Chemical Society.

In recent years, there is increasing evidence that the occurrence and development of cancer may have a non-negligible link with bacteria. Moreover, the suboptimal immunity of cancer patients may increase the chance of bacterial or fungal infections, leading to a high recurrence rate of cancer [150,151]. A photoactivated injectable and biodegradable hydrogel was designed by Han Huang et al. Bioactive glass nanoparticles (BGN-Fe-Ag_2_S) containing Ag_2_S nanodots conjugated to Fe were added to PEGDA and AIPH solutions to synthesize PBFA hydrogels [152]. This hydrogel had excellent photothermal properties for bacterial killing and also ablated tumors. Therefore, this hydrogel could be applied for anti-tumor purposes, could promote the healing of bacterially infected wounds, and could be used to prevent tumor recurrence and bacterial infection after tumor surgery. Antitumor hydrogels with the antibacterial agent Fe_3_O_4_ were prepared by free radical polymerization of oligo (oxyethylene) methacrylate. Norfloxacin (NOR), an antibacterial agent with antitumor activity, was then loaded into the hydrogel [153]. The hydrogel possessed both antibacterial and antitumor effects, but was non-toxic to normal cells and showed good biocompatibility. It had good potential in reducing the chance of bacterial infection in patients. For the treatment of skin tumors and bacterial infections, Hua Zheng et al. incorporated MoS_2_@MnFe_2_O_4_ nanocomposites into chitosan-grafted dihydrocaffeic acid (CS-DA) and GOx F127-CHO-loaded hydrogels to form injectable multi-responsive micellar nanocomposite hybrid hydrogels (CFMG) [154]. It could be used for bioenzymatic and photothermal enhanced chemotherapy (CDT) for skin tumors and bacterial invasion. Nádia S. V. Capanema et al. prepared AgNP@CMC-DOX nanocomplexes using DOX and Ag NP with carboxymethylcellulose (CMC) as a reducing agent and polymer ligand, which were then covalently cross-linked with citric acid to synthesize hybrid hydrogels (CMC@AgNP-DOX-CA) for the treatment of skin cancer and bacterial infections [155] (Figure 9). For the postoperative treatment of melanoma, Xiaoxuan Tang et al. prepared SFMA using filipin (SF) and methacrylate, and then introduced the photosensitizer Ce6 to form a photo-triggered in situ SFMA-Ce6 hydrogel system [156]. This hydrogel could convene macrophages and activate macrophages in the presence of NIR. The combination of PDT and immunotherapy was used to inhibit tumor recurrence and promote the healing of bacterially infected wounds.

## 5. Summary

In recent years, hydrogels have attracted considerable attention in the biomedical field because of their excellent biocompatibility, biodegradability, and on-demand drug delivery. Hydrogels can be used for antitumor or antibacterial therapy by loading chemotherapy or antibacterial drugs and combining PTT effect, PDT effect, SDT effect, etc. The treatment of tumors or bacterial infections by hydrogels can serve the purpose of treatment while effectively reducing the systemic toxicity of drugs to the body. For the application of hydrogels in tumor therapy, hydrogels mainly play the role of the carrier. They are used to destroy or inhibit tumors in situ by loading drugs, photothermal agents, photosensitizers, or acoustic sensitizers. They are delivered in a minimally invasive manner to the patient’s affected area and are able to fight the tumor more precisely. However, the current tumor models for antitumor hydrogels are mostly skin cancer or subcutaneous tumors, and it is difficult to apply antitumor hydrogels for internal human tumors. When hydrogels are used for the treatment of bacterial infections, they can not only be used as a carrier for antibacterial treatment, but also have their own antibacterial activity due to their own special structure. When hydrogels are used to treat tumors and bacterial infections simultaneously, they are mostly used in combination with phototherapy to prevent tumor recurrence and bacterial infections after tumor surgery. At present, hydrogels are still mainly used in wound dressing, drug delivery, and tissue repair. The application in tumor treatment is still in the experimental stage, and the clinical application requires the joint efforts of experts from various disciplines, such as material scientists, chemists, biologists, and doctors.

## Figures and Tables

**Table 1:** The classification of hydrogel functional components.

Function	The Classification of Function Component	Function Component
antitumor	chemotherapy drug	DOX [25,27,30,31,36,55,56,68,75,76,155], MTX [29], CD-CUR [30], RESV [31], ES [43], cisplatin [44,70], CA4 [51], Melittin [54]
	immune adjuvant	CTLA-4 [35] DPPA-1 [36], R837 [38], CpG ODN [46], Bestatin [56]
	phototherapy agent	PB [51], ICG [54,70,71,79] single-walled carbon nanotubes [55], Ag_2_S QD [56], Ce6 [67,156], PpIX [68], MB [74], AuNR [74,75], AgNR [75], black phosphorus quantum dots [76], BGN-Fe-Ag_2_S [152], Fe_3_O_4_ [153], MoS_2_@MnFe_2_O_4_ [154]
	acoustic sensitizer	TCPP [78], Fe_3_O_4_ [79]
antibacterial	organic or inorganic antibacterial agent	Ag NP [92,132,138,155], MoS_2_@PDA@Ag [96], CuS NP [97,123,141], AuNR [99], ZnO [101], Ag-ZnO [102], C/ZnO [103], Ag/Ag@AgCl/ZnO [104], Fe_3_O_4_/TiO_2_/Ag_3_PO_4_ [127], PEI@BPQD [133], THPS [134], BGN-Fe-Ag_2_S [152], Fe_3_O_4_ [153], MoS_2_@MnFe_2_O_4_ [154]
	antibiotic	gentamicin [106,107,116], chloramphenicol [109], erythromycin [111,112], vancomycin [114–116], NOR [153]
	inherently antibacterial hydrogel material	PDMAPS-co-PMA-Ade [117], chitosan [117,146,148,149,154], P(GMA-co-MPC) [118], MF-PEG [120], PCEC-QAS [121], IKFQFHFD peptide [7], RRRFRADA peptide [122], GIIKKIIKKIIKKI-GRADARADARADARADA-NH2 peptide [123], IKYLSVN peptide [126], PVA/PAM/PCBMAE [136]
	enzyme or nanoenzyme antimicrobial agent	GOx [126,127,154], MoS_2_@TA/Fe [129], Pt NP [130], lysozyme [146]

## Data Availability

Not applicable.
